# Psychoeducational intervention to improve oral assessment in people with autism spectrum disorder, BIO-BIO region, Chile

**DOI:** 10.4317/medoral.22560

**Published:** 2018-12-24

**Authors:** Lorena M. Orellana, Cecilia Cantero-Fuentealba, Lilian Schmidlin-Espinoza, Luis Luengo

**Affiliations:** 1 Special Care Dentistry Unit, School of Dentistry, Universidad de Concepción, Chile; 2 Department of Prevention and Dental Public Health, School of Dentistry, Universidad de Concepción, Chile

## Abstract

**Background:**

Lichen planus (LP) is a chronic autoimmune disease that affects the oral mucosa as well as the skin, genital mucosa and other sites. Objective: to evaluate the correlation between oral, genital and cutaneous lichen planus, in a sample of LP patients.

**Material and Methods:**

This descriptive study reviewed 274 clinical histories of patients, who all presented histological confirmation of lichen planus verified by a pathologist, attending research centers in Barcelona.

**Results:**

A total of 40 LP patients (14.59%) presented genital lesions. Of 131 patients with cutaneous LP (47.8%), the most commonly affected zones were the body’s flexor surfaces, representing 60.1% of cases. 24% of patients (n=55) related the start of the lesions with previous stress events. Of the 131 subjects with cutaneous lesions, 19% (n=25) also presented oral lichen planus (OLP). Of the total sample, 53.6% (n=147) of patients presented oral lesions. The systemic diseases most commonly associated with this patient sample were psychological problems such as stress, anxiety and depression (48%), hypertension (27%), gastric problems (12%), and diabetes (9.7%). A family history of lichen planus was found in only 2 cases (0,72%) out of the total of 274.

**Conclusions:**

Any patient with OLP should undergo a thorough history and examination to investigate potential extraoral manifestations. The fact that 37 patients with OLP in this series were identified with simultaneous involvement at more than one site highlights the need for exhaustive evaluation and multidisciplinary approaches to this disease.

** Key words:**Autism spectrum disorder, psychoeducational intervention, oral assessment, dentistry.

## Introduction

Within the population of patients requiring special care, those with autism spectrum disorder (ASD) are amongst the most challenging for dental staff (1,2). ASD is considered a developmental disorder and is usually characterized by deficits in communication and social interaction and by restrictive, repetitive, patterns of behavior, activities, and interests (3). ASD is treated using an interdisciplinary approach whose main objectives are to improve social communication skills and provide support to parents and families (4,5). Psychoeducational interventions are currently regarded as one of the most effective forms of treatment. They involve using a combination of psychological and educational approaches to improve the quality of life of people with ASD and focus on the specific characteristics and needs of this population (5-7).

Numerous studies have reported that people with ASD show disruptive behavior when receiving dental care (1,8-14). Oral examination involves opening of the oral cavity, something that people with ASD perceive as a serious threat. They may even feel that it is an aggressive intrusion and seek ways to protect that area of their body (15). This intense fear is, perhaps, the greatest challenge dentists face when treating these patients (13). All this makes the use of dental instruments and procedures extremely slow and complex (12) and as a result patients with ASD are frequently treated under general anesthesia (13,14,16,17) or with sedation (17-19). However, using certain techniques and strategies based on psychoeducational intervention models it may be possible to manage the behavioral, communicative and sensory alterations that patients with ASD may present when receiving dental assistance. The interventions used include behavioral and combined interventions, and those focused on communication and sensorimotor therapies (3,5,9-13,20).

Clinical rehearsals at home and in educational institutions are amongst the behavioral interventions used in dental care to anticipate and familiarize patients with basic dental instruments and procedures (12,15,21). The use of systematic desensitization and successive approaches that allow the patient to become familiar with the dental office, the dentist and the clinical procedures (9,15,22,23), and the use of Tell-Show-Do (T-S-D) or Tell-Show-Feel-Do (T-S-F-D) techniques, which consist of letting the person know in advance what procedures are going to be performed and what he/she is going to feel, have also been described (15,24,25). Other techniques are *in vivo* (9,15,26) or audiovisual modeling to improve compliance with clinical dental examination in people with ASD (9,15). Most of these techniques involve the use of reinforcement (8,9,15,21-23,25,26). The use of visual supports and the D-TERMINED program based on Applied Behavior Analysis (ABA) may also facilitate oral examinations and treatment (2,27). Combined interventions that have been used in dental care include some based on the TEACCH model (Treatment and Education of Autistic and Communication Handicapped Children) (15). Sensory interventions, such as SADE (Sensory-Adapted Dental Environment), have been employed to perform dental cleaning in a group of children with ASD (3). Communication-based interventions such as social stories, the Picture Exchange Communication System (PECS) and visual pedagogy have also been used to facilitate communication with children with ASD during dental treatment (1,3,10,11,15,20,24,28-30).

Several studies have used psychoeducational interventions for dental purposes (1-3,9-11,15,20,22-24,27-31), but most of these studies tested only one psychoeducational intervention technique and most involved children with ASD.

The aim of this study was to assess how effective a psychoeducational intervention program was in facilitating the performance of a series of steps of oral examination in children, adolescents and adults with ASD in the Bio-Bio region, Chile.

## Material and Methods

This was a prospective, quasi-experimental study involving pre-intervention, post-intervention and maintenance tests. The study was approved by the Ethics Committee of University of Concepción, Chile.

-Subjects

Eighteen educational centers and organizations for people with ASD from the Bio-Bio region, Chile were invited to participate in the study and 15 agreed to do so. They all had facilities where the intervention could be carried out and access to a psychologist or psychiatrist for diagnostic confirmation. These institutions belonged to various districts in the region: Agrupación Asperger Concepción (Concepción), Escuela Háblame de Amor (Concepción), Escuela Acercate a Mi Mundo (San Pedro de la Paz), Agrupación ASPI (San Pedro de la Paz), Agrupación AGANAT (Talcahuano), Escuela Especial María Ester Breve (Coronel), Escuela Especial Expresión de Amor (Tomé), Escuela Especial F-451 (Tomé), Asociación Hazme Parte de Tu Mundo (Tomé), Agrupación de Personas con Trastorno del Espectro Autista (Curanilahue), CET Paso a Pasito (Chillán), Escuela Especial Persevera (Chillán), Agrupación Mi Luna Azul (Chillán), Escuela Los Angelitos (Los Ángeles) and Escuela Especial ANTÚ (Los Ángeles).

**Figure 1:**
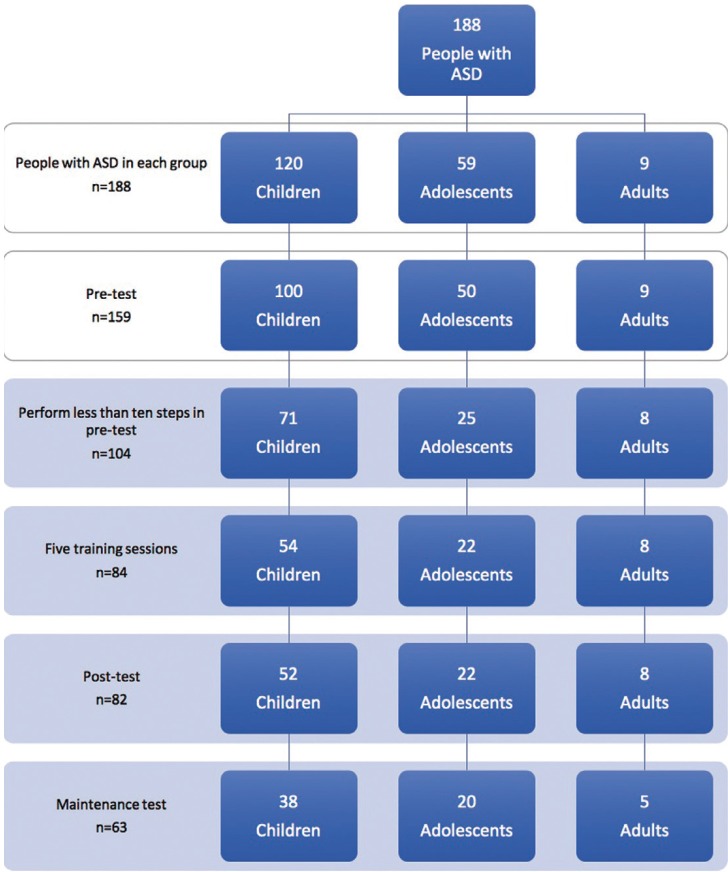
Flow chart of the study design by age group.

A total of 188 people with ASD from these institutions, diagnosed with autistic disorder, Asperger’s syndrome or Pervasive Developmental Disorder-Not Otherwise Specified (PDD-NOS), agreed to participate in the study and provided written, informed consent. Only 104 met all the following inclusion criteria: a) aged four years or older; and b) completed fewer than 10 steps of dental examination in the pre-test (Fig. 1).

-Procedure

Meetings were held in all the participating institutions to explain the aims of the intervention to the parents and caregivers of subjects. Those who agreed to participate were asked to provide written consent. In following sessions a social worker filled out a questionnaire on the family, dental, sensory and behavioral background of each subject with ASD and a psychologist (A) evaluated the social maturity of people with ASD using a Chilean version of the Vineland Social Maturity Scale, (E/C) (2012).

Two pediatric dentists with vast experience in the care of patients with special needs participated in the intervention. They had previously carried out a pilot study in two institutions of the Bio-Bio region (2015).

Eight sessions were carried out. The first one constituted the pre-intervention test: behavior and the number of completed progressive steps of dental examination were measured. These steps were: 1º Entering the room, 2º Sitting down in the dental chair, 3º Lying back in the dental chair, 4º Tolerating direct light on the face, 5º Opening the mouth, 6º Tolerating manipulation of the mouth with gloves, 7º Examination with the mouth mirror, 8º Examination with the probe, 9º Examination with the mirror and probe, and 10º Examination of dental occlusion. Subsequently all subjects received 5 individual psychoeducational sessions. These sessions were held weekly in a specific room in each participating institution and lasted 10 to 15 minutes. The patient always saw the same dentist and accompanied by an assistant. The seventh session was a post-intervention test and the final session, carried out one month later constituted the maintenance test; behavior during examination and the number of completed examination steps were assessed. All these activities were carried out in a room equipped to resemble a dental office, with a dental chair, seat, table, portable lamp, dental materials and instruments (for details of the intervention, see (15)).

During the pre-intervention test the dentist asked the subject to submit to the 10 steps of the dental examination but did not force him or her to comply. The dentist explained the steps using a sequence of pictographic images; this activity was filmed by the assistant. In the following 5 sessions were structured as summarized below:

1st Session: Through successive approaches each subject was allowed to get familiar with the new environment of the simulated dental office, interact with the dentist and become familiar with the dental instruments. Later in the session the techniques of T-S-F and visual pedagogy were used.

2nd Session: Audiovisual modeling was employed to demonstrate the 10 steps of the oral examination. A tablet computer was used to play the subjects a 2.75 3.75-minute video showing each of the 10 steps twice. Successive approaches, T-S-F-D and visual pedagogy were also used when necessary.

3rd Session: An educator or a classmate from the same center who had submitted to the 10 steps in the pre-test served as a model for in vivo modeling of the steps. Each examination step was modeled twice. Successive approaches, T-S-F-D and visual pedagogy were used when necessary. Subjects were also encouraged to participate in a role-playing activity.

4th Session: Behavioral training involving undergoing each of the 10 steps of the oral examination sequentially was carried out. Each step was performed twice with real objects such as the oral mirror and probe. Successive approaches, visual pedagogy and/or the T-S-F-D technique were used, if necessary.

5th Session: Self-modeling was used: subjects learned behavioral patterns by observing their own performance in the previous session. To make this possible subjects were photographed performing the target behaviors and the images were edited to remove undesirable elements.

Sessions were carried out in a playful and warm atmosphere. Verbal instructions were used along with positive reinforcement tailored to the interests of each subject. Puppets, dolls and a tablet computer were used with the children. Adolescents were given the option of using puppets/dolls or the dental macro model and the tablet computer. The dental macro model and the tablet computer were used with adults.

The dentist carried out the post-intervention test in the seventh session, after the five intervention sessions. The test was repeated one month later, in the eighth session to assess persistence of the acquired learning. All three tests (pre- and post-intervention; maintenance) were filmed for later assessment by a psychologist (B), who evaluated the number of steps completed (1-10) and the behavior of the subject (using Frankl’s scale: 1-4). Subsequently all the subjects underwent a clinical dental examination.

-Data analysis

Univariate and bivariate descriptive statistics (mean; standard deviation) were calculated for quantitative variables and relative frequencies for categorical variables. Paired t-tests and Wilcoxon tests were used to compare the number of steps completed and the behavior, respectively, in the pre- and post-intervention performance in each age group. Line graphs with markers were used to visualize the results. Differences were considered significant at the 5% level. Data were analyzed with the statistical software SPSS-23.

## Results

Eighty-two of the 104 people with ASD who met the eligibility criteria completed the psychoeducational intervention and pre- and post-intervention tests (male n = 69; female n = 13). This sample had a mean age of 9.1 years (SD = 4.9, range: 4-21.2) ( Table 1). The majority of people with ASD were diagnosed with autistic disorder (69.5%), of these, 49.1% had moderate intellectual disability, and 35.1% severe intellectual disability.

**Table 1:**
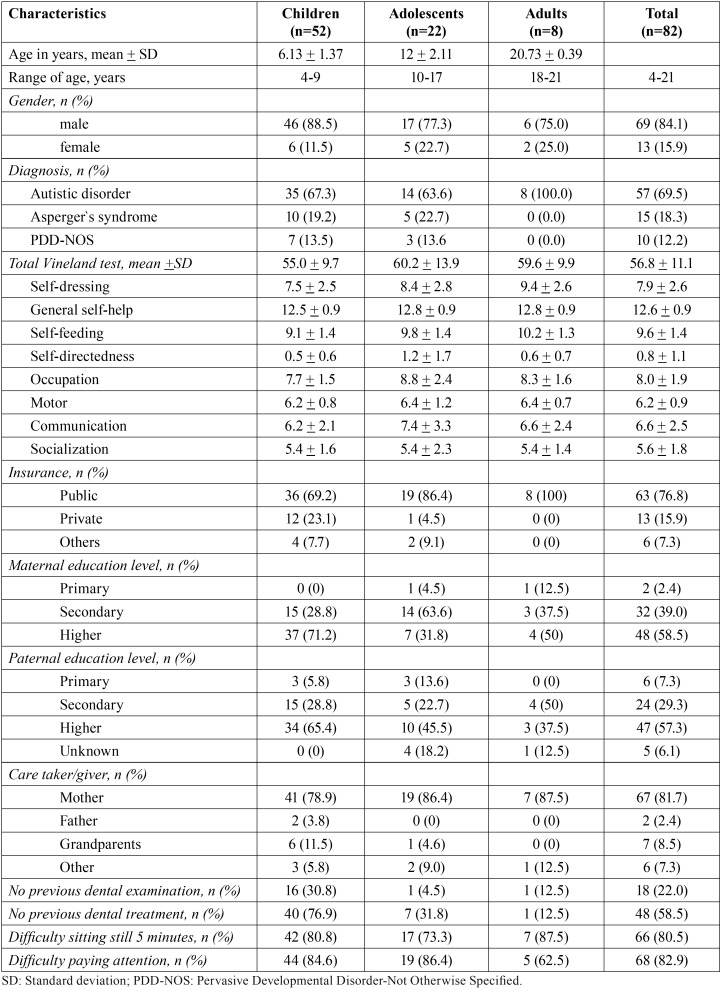
Descriptive characteristics of subjects by age group.

**Figure 2:**
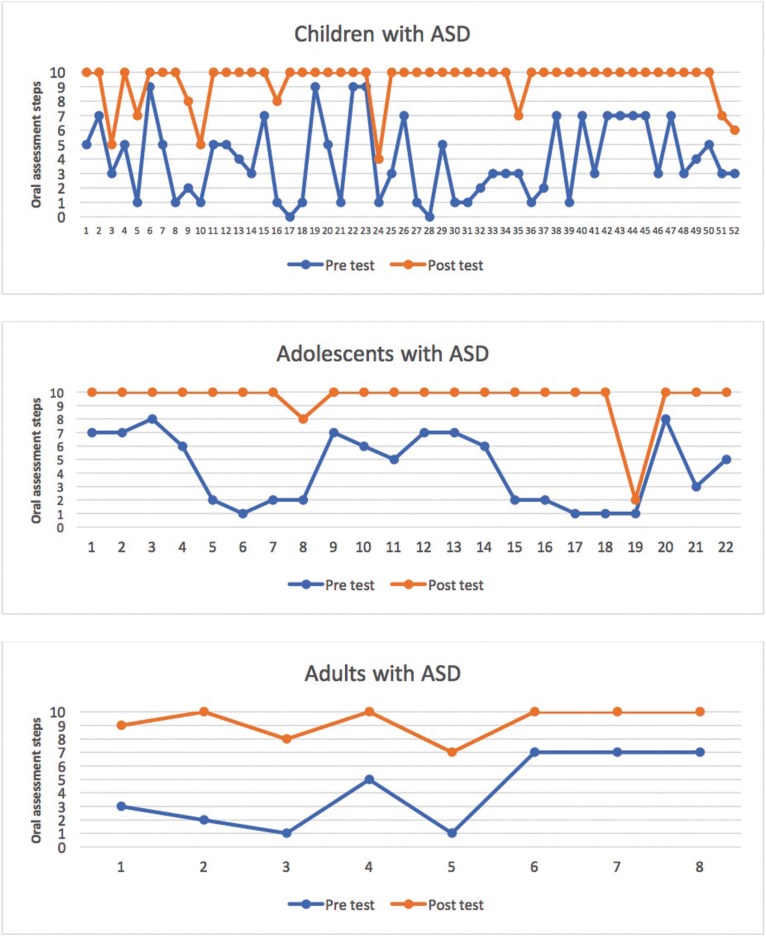
Comparison by age group of number of oral assessment steps completed pre- and post-intervention.

The sample of 82 people with ASD who took part in the intervention completed a mean of 4.1 steps (SD = 2.6) in the pre-intervention test and 9.4 (SD = 1.5) in the post-intervention test, resulting in a difference of 5.3 steps (*p* < 0.0001, t-test), and a Cohen´s d effect size of 2.5. The increases in steps completed were 5.4, 5.2 and 5.1 in children, adolescents and adults respectively, and the increase was significant in all groups ( Table 2a, Fig. 2).

**Table 2:**
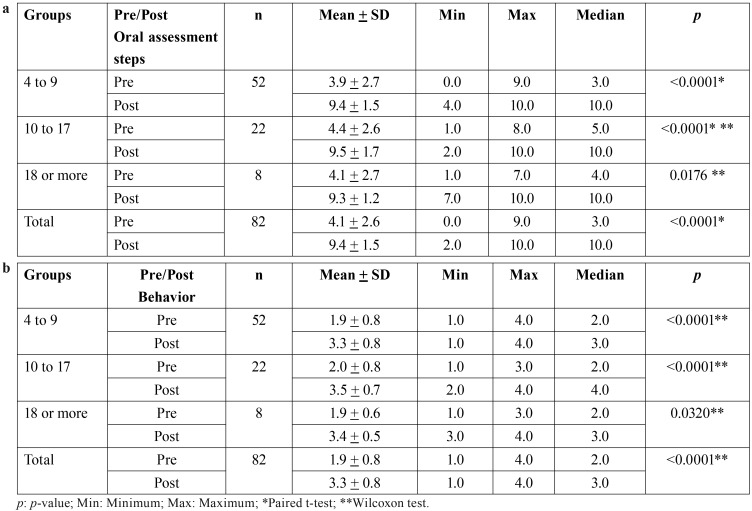
Comparison of pre- and post-intervention performance by age group: number of oral assessment steps completed (a) and Frankl scale behavior score (b).

According to the diagnosis, the mean of steps during the pre-intervention was 6.3 steps in people with Asperger syndrome, 4.0 steps in people with PDD-NOS, and 3.5 steps in those diagnosed with autistic disorder. The increase in steps was 3.7 in the group of people with Asperger’s syndrome, 5.8 in people with PDD-NOS, and 5.7 in people with autistic disorder. We also compared the increase in steps by previous dental experience: with respect to oral examination and dental treatment. We observed differences of 0.3 steps between the subjects without experience (5.6) and those with previous experience (5.3) in dental examination. A difference of 1.1 steps was found between the subjects without prior experience in dental treatment (5.7) and those with prior experience in dental treatment (4.8), of which 88.2% had been treated under sedation and 67.6% under general anesthesia.

**Figure 3:**
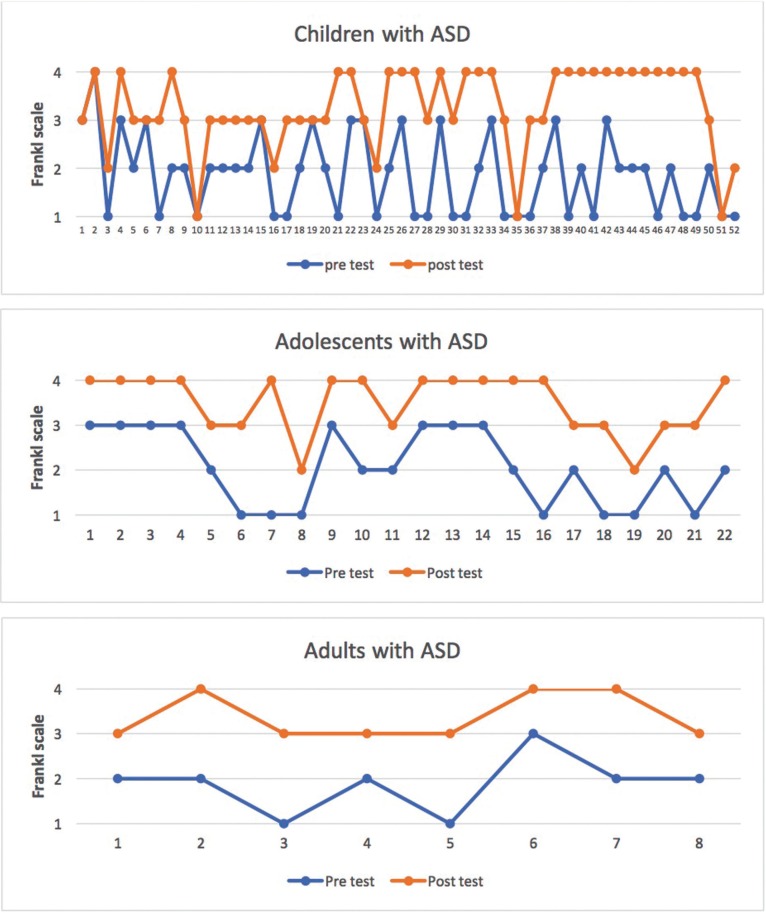
Comparison by age group of number of Frankl Scale scores pre- and post-intervention.

The median pre-intervention Frankl behavioral score was 2 (negative behavior); post-intervention this increased to 3 (positive behavior) and the improvement was statistically significant (*p* < 0.0001, Wilcoxon test). Behavior improved by one Frankl category in children and adults and by two in adolescents ( Table 2b, Fig. 3).

Only 63 out of 82 subjects underwent the maintenance test. The results indicated that the number of examination steps completed was similar at the post-intervention (M = 9.6, SD = 1.3) and maintenance tests (M = 9.7, SD = 1.2). Regarding the comparison of steps between post-intervention and maintenance tests by age groups, no significant variation was found either ( Table 3a). The median Frankl behavioral score in the post-intervention and maintenance tests was 4 (very positive behavior). Separate comparisons for each age group showed that behavior was similar at post-intervention and maintenance tests in adolescents and adults, but improved by category in children ( Table 3b).

**Table 3:**
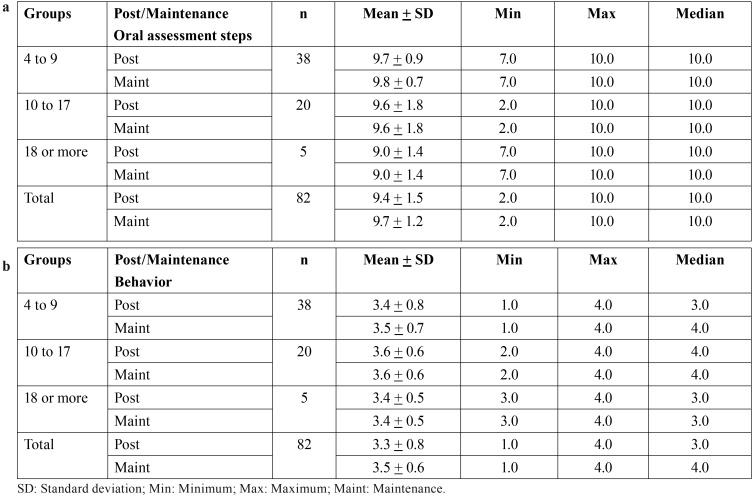
Comparison of post-intervention and maintenance test performance by age group: oral assessment steps completed (a) and Frankl scale behavior score (b).

## Discussion

Providing dental care to people with ASD is extremely challenging and so a high percentage of patients are usually treated under general anesthesia (1,3,13,14,17). There are, however, some techniques and psychoeducational strategies that facilitate dental procedures, such as oral examination, and can be used to reduce the excessive use of general anesthesia with these patients. The population of people with ASD is very heterogeneous and so it is very important to use flexible interventions involving multiple techniques when working with it. The intervention used in this study was effective, producing an increase of more than 5 in the number of steps completed. Frankl behavior ratings also improved (from negative to positive) in this sample of Chilean children, adolescents and adults with ASD.

Our sample of people with ASD was larger than the samples in many other studies of psychoeducational intervention targeting dental procedures (1-3,9-11,15,20,24,29,30). No previous studies have attempted to assess groups of children, adolescents and adults with ASD; most have only assessed children (1,3,9,10,23,24,27,30) or have assessed children and adolescents (2,22,28,29,31) without differentiating between them, which is important as hormonal changes in adolescence could influence the results (15). Thus, the present study makes a significant contribution to knowledge because we analyzed a group of adolescents with ASD, as did an Indonesian study (20). Current studies in the literature use different psychoeducational methods and outcome measures, which makes it very difficult to make comparisons between populations of people with ASD.

After the five psychoeducational sessions 82.9% of the subjects were able to complete the last step of the oral examination, which was to keep the dental arches together; this figure is somewhat higher than the 77.7% observed by Orellana *et al.* (15), but the percentage of people with ASD who completed the penultimate step, toleration of the intraoral use of the dental mirror and caries probe (84.1%) was lower than the 90.3% observed by Orellana *et al.* (15). Looking separately at the performance of the child and adult groups in both studies reveals that the percentage of children completing all ten steps was higher in the present study (82.7% vs. 65.8%) whereas the percentage of adults completing all ten steps was higher in the Orellana *et al.* (15) (91.2% vs. 62.5%). Looking at the percentage of children completing the seventh step (toleration of intraoral use of dental mirror), the figure in the present study (92.3%) was similar to the 92.1% reported by Orellana *et al.* (15), and higher than the 70% and 81.3% observed respectively by Nilchian, Shakibaei and Jarah (30) and Bäckman and Pilebro (10) after visual pedagogy. When comparing the effect of the intervention, according to the diagnosis of people with ASD, we observed that it was higher in people with PDD-NOS and autism disorder than in people with Asperger`s syndrome; however, the latter had a higher number of steps completed in the pre-intervention, which may be due to the greater degree of functionality people with Asperger`s syndrome usually have. When comparing the effect of the intervention according to the previous dental experience, it was observed that the increase in steps in the examination and in the previous dental treatment were similar.

In the present study, a high percentage of the people with ASD who underwent previous dental treatment had been treated under sedation and/or general anesthesia, these figures were higher than those reported by others authors (1,2,17,19).

Pre-intervention 75.0% of the children with ASD displayed negative behavior (Frankl score = 2) or very negative behavior (Frankl score = 1), which is similar to the 73.7% reported by Orellana *et al.* (15); however 87.5% of the adults in this study displayed negative or very negative behavior pre-intervention, which is a much higher figure than the 67.6% in the study by Orellana *et al.* (15). At the post-intervention test 86.5% of the children and 100% of the adults in the present study displayed positive behavior (Frankl score = 3) or very positive behavior (Frankl score = 4), figures which are very similar to those reported by Orellana *et al.* (15) (81.6% and 100%, respectively).

An important aspect of this study was the inclusion of a maintenance test to determine if gains were maintained for one month after the intervention. We found that the improvement in number of steps completed was maintained (post-intervention: M = 9.6, maintenance: M = 9.7). There is no previous research including this variable.

If an intervention for people with ASD is to be successful then collaboration with parents or caregivers is essential. They must ensure that the person with ASD attends intervention sessions regularly, so that he or she becomes familiar with dental care procedures and they become part of his or her routines, as this will minimize disruptive behavior.

The limitations of this study include the relatively small sample of adults with ASD. This is because there are few adults in Chilean institutions for people with ASD, reflecting a lack of support for this age group. It is important, therefore, to create more day centers for adults with ASD in order to improve their quality of life and social inclusion. Another limitation was the fact that we did not measure the behavior and number of steps completed in each session because it exceeded the study budget.

The results of this study, carried out in Chile, are comparable to those obtained in Spain by Orellana *et al.* (15), confirming that this type of intervention has a positive impact on toleration of clinical dental examination in people of all ages with ASD, different degrees of severity, and from different locations.

The proposed psychoeducational intervention program involves the mastery of simple psychoeducational techniques that can be used by a dentist in a dental clinic to facilitate oral examination. It is the initial step to establish an appropriate diagnosis and propose a treatment plan, so these patients can be treated in the dental clinic and, in this way, avoid unwanted behaviours that could seriously hinder their treatment.
